# Mir-183 functions as an oncogene via decreasing PTEN in breast cancer cells

**DOI:** 10.1038/s41598-023-35059-x

**Published:** 2023-05-19

**Authors:** Samaneh Mohammaddoust, Majid Sadeghizadeh

**Affiliations:** Genetics Department, Faculty of Biological Sciences, Tarbiat Modares University, Tehran, Iran

**Keywords:** Cancer, Cell biology, Genetics, Molecular biology, Diseases, Medical research

## Abstract

Regarding the important role of microRNAs in breast cancer, investigating the molecular mechanisms of miRs and their impacts on breast cancer progression is critical. Thus, the present work aimed to investigate the molecular mechanism of miR-183 in breast cancer. PTEN was validated by dual luciferase assay as a target gene of miR-183. Through qRT-PCR analysis, miR-183 and PTEN mRNA levels in breast cancer cell lines were measured. To determine the impacts of miR-183 on cell viability, the MTT assay was used. Moreover, flowcytometry was applied to analyze the effects of miR-183 on the cell cycle progression. To detect the effects of miR-183 on the migration of BC cell lines, wound healing was used along with a Trans-well migration assay. Western blot was utilized to assess the effect of miR-183 on PTEN protein expression. MiR-183 can exert an oncogenic effect by promoting cell viability, migration, and cell cycle progression. It was revealed that cellular oncogenicity is positively regulated by miR-183 by inhibiting the expression of PTEN. According to the present data, miR-183 may play a vital role in the progression of breast cancer by reducing PTEN expression. It may be also a potential therapeutic target for this disease.

## Introduction

Breast cancer (BC) is the most identified cancer among women. It accounts for 11.7% of all diagnosed cancers with new cases of about 2.3 million^1^. Despite the recent improvement in the approaches for the treatment and screening of breast cancer^2^, the molecular pathogenesis of breast cancer is still unclear making breast cancer a main public health challenge^3^.

MicroRNAs (miRNAs) are single-stranded, endogenously expressed, short, noncoding RNAs with a length of 18–25 nucleotides. According to several studies, miRNAs have main roles in differentiation, regulation of cellular growth, apoptosis, and proliferation^4^. Moreover, it was revealed that miRNAs can act as tumor suppressors or oncogenes. They are involved in different biological processes including tumor progression, tumor initiation, and drug resistance in different kinds of cancer^5–10^. Several microRNAs have been recognized as therapeutic targets, biomarkers, and prognostic markers for breast cancer^11^.

BC-related differentially expressed microRNAs (DE-miRs) were recognized and assessed in the present study. We mainly concentrated on the PI3K/AKT pathway, which is one of the most vital signaling paths included in the growth and control of tumors in breast cancer. We selected miR-183 for study among the considerably dys-regulated microRNAs in breast cancer. The reason is that according to Bioinformatics analysis, the 3-UTR of PTEN, a key gene in the PI3K/AKT signaling path, can be targeted by miR-183.

Phosphatase and tensin homolog (PTEN) is well recognized as a tumor suppressor gene. It was mutated frequently or deleted in various cancers, such as prostate cancer, small-cell lung cancer, endometrial cancer, etc.^12^. Depowski et al. also revealed that PTEN insufficiency was related to breast cancer poor prognosis^13^. PTEN insufficiency promotes the PI3K/AKT path, which is a vital signal for the survival of cancer cells. Therefore, specific inhibitors targeting proteins of the PI3K/AKT pathway are now being evaluated in clinical trials as a new approach against breast cancer^14^. It has been proven that the miR-183 expression is up-regulated in tumor tissues and patient serum in BC, which indicates its possibly an oncogenic role^15,16^.

In the present study, the effect of miR-183 on cell viability, cell cycle progression, and migration was investigated, and the interaction between miR-183 and PTEN in BC cells was examined.

## Result

### Differentially expressed microRNAs in breast cancer samples and the expression profile of miR-183 and PTEN in BC cell lines

After selecting the data sets based on our criteria, quality control, and normalization, each data set of the microRNAs expression profile was analyzed. The P value less than 0.05 and a basemean of 100 or higher were set as the DE microRNAs cut-off values. The common DE-microRNAs between the two study data were recognized. Ultimately, miR-183 among common DE-microRNAs was chosen for assessment.

**Figure 1:**
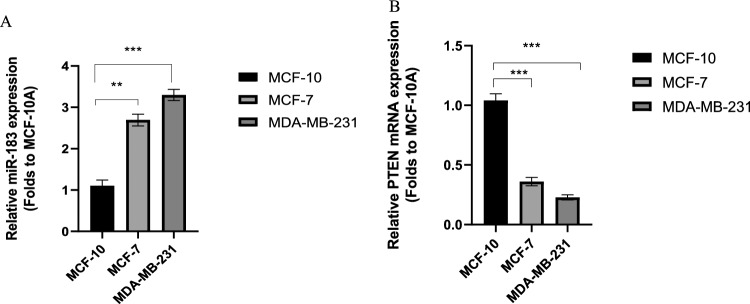
The relative expression of miR-183 and PTEN in MCF-7, MDA-MB-231, and MCF-10A cells was measured with qRT-PCR (**A**) Relative expression of miR-183; **p = 0.0024, ***p = 0.0009 (**B**) Relative expression of PTEN; ***p = 0.0008, ***p = 0.0004. Data are presented as the mean ± SD. *P < 0.05; **P < 0.01; ***P < 0.001.

To determine the expression of miR-183 in MCF-7 and MDA-MB-231 cells, it was compared with MCF-10A, a normal breast epithelial cell line. The evidence revealed that miR-183 was increased in MCF-7 and MDA-MB-231cells compared to MCF-10A cells (Fig. 1A, p = 0.0024, p = 0.0009). To assess the levels of PTEN in cell lines in the present study, qRT-PCR was used. According to the data, PTEN expression in the BC cell lines was considerably lower in comparison to the MCF10 cells (Fig. 1B, p = 0.0008, p = 0.0004).

### PTEN is a direct target gene of miR-183

**Figure 2:**
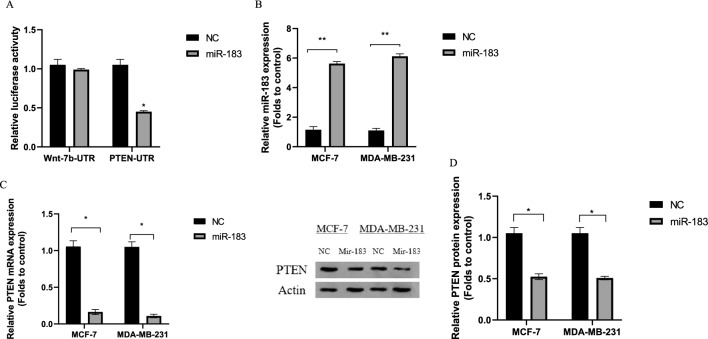
MiR-183 targets PTEN expression. (**A**) PTEN is a direct target of miR-183 demonstrated by 3UTR luciferase assay; *p = 0.0451; Wnt-7b gene 3′UTR sequence was used as off target in this experiment. (**B**) The transfection efficiency of miR-183 was measured by qRT-PCR 48 h after transfection **p = 0.0032, **p = 0.0012 (**C**) The mRNA of PTEN was measured by Real-time PCR in MCF-7 and MDA-MB-231 transfected with miR-183; *p = 0.0167, *p = 0.0200 (**D**) The protein expression of PTEN was measured by Western blot assay in MCF-7 and MDA-MB-231 transfected with miR-183; *p = 0.0273, *p = 0.0420. A full-length image is included in a Supplementary Information file. *NC* negative control. *p < 0.05; **p < 0.01; ***p < 0.001.

PTEN is a potential target mRNA of miR-183 according to miRNA target analysis algorithms (miRdb, Diana, RNAhybride, and starbase). Thus, PTEN was chosen for further examination. To clarify whether PTEN was a direct target of miR-183, luciferase reporter constructs were integrated with the 3′-UTR of PTEN mRNA comprising the miR-183 binding sites. 3′UTR sequence of Wnt-7b was used as an off-target control. According to the luciferase reporter assay, in the PTEN 3′-UTR group that was transfected with miR-183, the luciferase activity was considerably reduced compared to the off-target control groups (3′UTR sequence of Wnt-7b construct) (Fig. 2A, p = 0.0451).

The transfection efficiency of miR-183 in breast cancer cell lines was elucidated by the transfection of MDA-MB-231 and MCF-7 cells with miR-183 indicating enhanced levels of miR-183 (Fig. 2B, p = 0.0032, p = 0.0012). Moreover, RT-qPCR and western blotting analyses were performed after transfection to detect the expressions of PTEN. The mRNA and protein expressions of PTEN were suppressed significantly in cells transfected with miR-183 in comparison with the control group (Fig. 2C, p = 0.0167, p = 0.0200 and Fig. 2D, p = 0.0273, p = 0.0420).

### MiR-183 improves the cell migration ability of MCF7 and MDA-MB-231 cells

**Figure 3:**
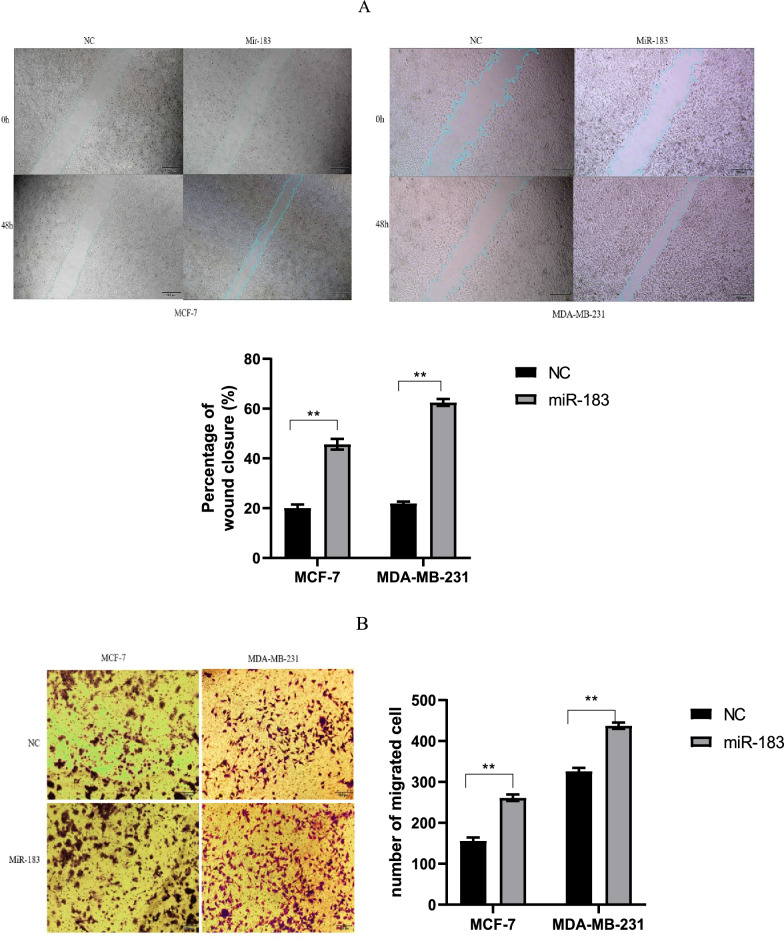
Overexpression of miR-183 leads to considerable enhancements in cell migration. (**A**) Wound healing assay; **p = 0.0084, **p = 0.0038 (**B**) Trans-well assay; **p = 0.0054, **p = 0.0049. *p < 0.05; **p < 0.01; ***p < 0.001. *NC* negative control.

Through trans-well and wound healing assays, the role of miR-183 in the migration of BC cells was explored to assess the migration capability of MDA-MB-231 and MCF7 cells after transfection with miR-183. According to the wound healing assay, there was less migration for the cells in the control group compared to the miR-183 group (Fig. 3A, p = 0.0084, p = 0.0038). Moreover, the trans-well assay revealed that cell migration was enhanced by miR-183 up-regulation in both MDA-MB-231 and MCF7 cells (Fig. 3B, p = 0.0054, p = 0.0049). Thus, based on our observation, the BC cells’ migration ability was promoted by miR-183.

### MiR-183 could induce cell viability and cell cycle progression of BC cells

**Figure 4:**
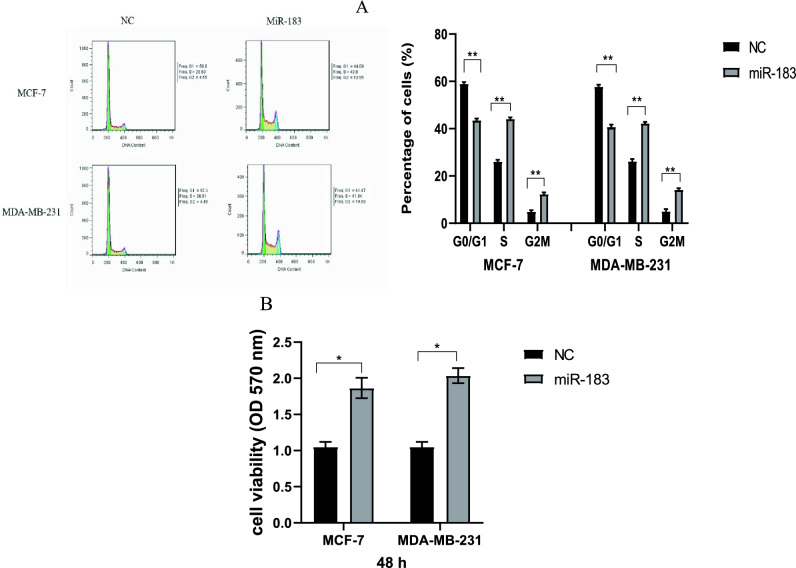
The effect of miR-183 on cell cycle progression and cell viability in breast cancer cells. (**A**) Cell cycle profiles were analyzed using flowcytometry after MCF-7 and MDA-MB-231 cells transfection with miR-183; (G0/G1: **p = 0.0019, **p = 0.0026; S: **p = 0.0012, **p = 0.0074; G2/M: **p = 0.0068, **p = 0.0093). (**B**) Cell growth viability was measured by MTT assay 48 h after transfection; *p = 0.0395, *p = 0.0127. The un-transfected cells serve as the negative control. *p < 0.05; **p < 0.01; ***p < 0.001. *NC* negative control.

To assess the mechanism of promoting cell proliferation by miR-183, the effects of miR-183 on cell cycle regulation were determined in the present work. In this regard, MDA-MB-231 and MCF-7 cells were transfected with miR-183. Then, flowcytometry was used to analyze the cell cycle status 48 h after transfection. The number of cells in the G0/G1 phase was decreased in BC cells transfected with miR-183. However, the number of cells increased in the S and G2/M phases (Fig. 4A, G0/G1: p = 0.0019, p = 0.0026; S: p = 0.0012, p = 0.0074; G2/M: p = 0.0068, p = 0.0093). Therefore, miR-183 may contribute to the enhancement of cell cycle progression in breast cancer cells.

The effects of miR-183 on cell viability were investigated through an MTT assay. According to the results, the viability of MCF7 and MDA-MB-231 cells was increased by the miR-183 overexpression (Fig. 4B, p = 0.0395, p = 0.0127). Therefore, it was revealed that miR-183 had a key role in the BC cells’ viability.

## Discussion

Several reports have shown the dysregulation of microRNAs in the early stages and development of human cancers. Considering the dysregulated expression of microRNAs in cancers, the regulation of cellular microRNA levels may be developed as a potential therapeutic approach.

Our analysis of two previous RNAseq studies (GSE117452, GSE68085) revealed that various microRNAs were significantly up-regulated in tissues from breast cancer patients compared to tissues from healthy individuals. Mainly, we concentrated on the PI3K/AKT path as one of the most vital signaling pathways included in breast cancer. Thus, miR-183 was selected from the list including microRNAs with dysregulated expression. The reason is that miR-183 can target PTEN, a key gene in the PI3K/AKT pathway, based on the bioinformatics analysis. Furthermore, according to a former work, PTEN can potentially target miR-183 in lung cancer^20^. In this regard, our study further proved that miR-183 can directly target PTEN via the miR-183 binding site at the 3′-UTR in breast cancer.

PTEN has a key role in tumor growth, metastasis, and invasion. PTEN can restrict survival and growth signals through the limitation of the PI3K/AKT pathway activity. By a reduction in functional PTEN, constitutive activation of downstream components of the PI3K/AKT pathway including Akt is caused, thus resulting in metastasis and tumor progression^21^. Therefore, down-regulation of PTEN by miR-183 may have a role in the transformation and enhanced tumor cell survival.

It is suggested that miR-183 is included in the BC pathogenesis. Moreover, it may be a biomarker for diagnosing and treating BC. Therefore, in this study the biological impact of miR-183 and the molecular mechanisms by which miR-183 modulates the behavior of breast cancer cells was investigated.

Considering the previous results, it was indicated that miR-183 might stimulate BC progression, thus revealing miR-183 as a cancer promoter. It was revealed that miR-183 overexpression led to the down-regulation of PTEN in the breast cancer cells. Our investigation was also performed on PTEN expression at the protein and mRNA levels in the MDA-MB-231 and MCF7 cells after transfection with miR-183. The findings revealed that by miR-183 overexpression in MCF7 and MDA-MB-231 cells, the expression of PTEN in mRNA and protein levels was down-regulated compared to the control group.

Moreover, MDA-MB-231 and MCF7 were utilized as an in vitro model for examining the miR-183 overexpression functional impact on cell features. The Trans-well and wound-healing assays were used to assess the migration of the breast cancer cells after miR-183 overexpression. According to the results, more MDA-MB-231 and MCF7 cells migrated in the miR-183 transfection group compared to the control group. Based on the cell cycle analysis, the number of cells in the G2 and S stages increased by transfection of the cells with miR-183. Thus, miR-183 enhanced the migration and proliferation of breast cancer cells. Moreover, the results of a luciferase reporter assay indicated that PTEN was a target gene of miR-183. The protein expression analyses of PTEN in the miR-183-treated BC cell lines further supported these results.

Similar reports were provided in various cancers. For instance, it has been shown that in several types of cancer, including gastric and colon cancer, miR-183 is overexpressed and acts as an oncogene^22–24^. It was also indicated that miR-183 promotes tumor development in pancreatic, prostate, and non-small cell lung cancers^20,25,26^. Macedo et al. found that miR-183 may play the role of an oncogene by targeting RB1 protein in MDA-MB-231 cells^27^. Also, miR-183 exerts oncogenic effects in breast cancer and can play an important role in the field of using anti-miRs as a cancer treatment^28^. Previously, it was revealed that cell proliferation, angiogenesis, and metastasis were promoted by miR-183 through negative regulation of FHL1 in BC^29^. Nevertheless, it might also act as a tumor suppressor in some cancer types including cervical cancer^30^. Tumor differentiation, metastasis, and invasion are accelerated by miR-183^31,32^. It is an important microRNA involved in cellular processes in cancers. According to Wang et al., PTEN is restrained by miR-183 resulting in the promotion of lung cancer^20^.

As we know, this is the first study to prove the regulation of breast cancer by the miR-183/PTEN pathway. It suggests that the miR-183/PTEN pathway can be considered a possible effective target in the treatment of breast cancer. It previously was reported that PTEN may act as a dual specificity phosphatase thus regulating cell growth, invasion, apoptosis, and differentiation by regulating the PI3K/AKT signaling pathway negatively. It has been known that this pathway plays a main role in several cellular functions such as adhesion, proliferation, migration, angiogenesis, metabolism, and invasion^33–35^.

According to our data, miR-183 can increase cell viability, accelerate cell cycle progression, and induce further migration of BC cell lines by down-regulation of PTEN. Actually, in this study, it was revealed that malignant phenotypes might be promoted by miR-183 in BC. Thus, it can be considered as a potential biomarker for early diagnosis and treatment of BC. Considering that a microRNA can target more than 100 genes^36^, it is expected that there are additional targets of miR-183 that have not yet been identified in different cellular systems. These genes may also contribute to metastasis and invasion. Therefore, our study shows that miR-183 targeting may be a suitable approach to prevent tumor metastasis.

## Conclusions

It can be concluded that miR-183 serves as an oncogene by regulating the PI3K/AKT signaling path. Mir-183 up-regulation can promote malignant phenotypes, including migration, cell cycle progression, and proliferation of BC cells by down-regulation of PTEN protein expression and then, PIK pathway progression. Therefore, with these results, the molecular mechanisms of miR-183 in breast cancer can be comprehended, thus providing valuable insights into the clinical use of miR-183 in such cancers. Indeed, the inhibition of miR-183 expression can reduce the malignant phenotype and inhibit the proliferation of cancer cells.

## Materials and methods

### In silico analysis

#### Differentially expressed microRNAs in breast cancer by high-throughput data

Based on our criteria, we looked for gene expression studies from the NCBI-GEO database. The criteria included (1) original studies between breast cancer and healthy humans and (2) the type of dataset, which was microRNA expression profiling by high-throughput sequencing. There are rare high-throughput data on microRNAs in breast cancer. Thus, we gathered only a small number of RNA-Seq datasets in GEO assessing microRNA expression in healthy controls and BC patients. To identify differentially expressed miRNAs, the Galaxy-use online database was used, which is a web-based, open platform for biomedical research with intensive data^17–19^. The selected datasets was imported to Galaxyuse for further analysis. RNA-Seq data was quality controlled using the FastQC software. RNA-Seq data with weak quality controls were excluded. Ultimately, two gene expression datasets were chosen (GSE68085 and GSE117452) using specimens from healthy humans and breast cancer tissue. Then, the Trimgalore software was used to trim and remove unwanted biases from the RNA-Seq data. Again, the quality control (QC) of trimgalore output was checked using FastQC software. We used HISAT2 to alignment RNASeq reads (fastq file) to the reference genome (BAM file format was resulted). Then, we obtained the annotation file (GTF format) from UCSC browser and import it to Galaxyuse. To count the aligned reads HTSeq software was used. Finally, the count normalization and analysis was performed by DESeq2. The DESeq2 software was used to analyze and identify miRNAs in the RNA-Seq data. In this section, the expression levels of miRNAs were compared between cancer and normal Tissue. Our standards for evaluation of the miRNAs expression changes were log2 (FC) > 2, p-value < 0.05 and a base mean of 100 or higher. The common DE-microRNAs between the two study data were recognized. Ultimately, miR-183 among common DE-microRNAs was chosen for assessment. Based on the bioinformatics results (GEO database: GSE68085 and GSE117452), a high level of miR-183 was found in breast tumor samples. DIANA-miRPath indicated one of the crucial signaling pathways implicated in breast cancer cells is PI3K/AKT. Thus, we mainly concentrated on the PI3K/AKT pathway, which is one of the most vital signaling paths included in the growth and control of tumors in breast cancer. In total, we concentrated on the microRNAs that are able to target key genes in the PI3K/AKT signaling path. Therefore, miR-183 was chosen for study among the considerably dys-regulated microRNAs in breast cancer, because according to miRNA target analysis algorithms (miRdb, Diana, RNAhybride, and starbase), the 3′-UTR of PTEN, a key gene in the PI3K/AKT signaling path, can be targeted by miR-183. Therefore, mir-183 may promote PI3K signaling pathway through the PTEN suppression in breast cancer cells. It was therefore selected for experimental research.

### Cell culture and transfection

The Pasteur Institute of Iran presented human breast cancer cell lines (MCF-7, MDA-MB-231), HEK-293T, and MCF-10A. Cells were cultured in DMEM media (high glucose; Invitrogen) supplemented with antibiotics and 10% fetal bovine serum (Gibco). The growth conditions for the cells were a humid environment at 37 °C and 5% CO_2_. To meet the demand for various assays, proper cell numbers were seeded. A Turbofect transfection reagent (Thermo Fisher Scientific) was used to transfect the plasmids based on the instructions of the manufacturer. The transfected cells were utilized for further assessments.

### DNA constructs and related restriction enzymes

Specific primers carrying XhoI and HindIII (Thermo Fisher Scientific) restriction enzyme sites were used by amplifying the sequence of miR-183 genes along with its flanking sequence from genomic DNA by PCR. The purified PCR product associated with miR-183 was digested with related restriction enzymes and incorporated into the pEGFP-N1 vector (Promega). Furthermore, PCR was used to amplify the 3′-UTR segments of the PTEN containing the binding site miR-183. Using NotI and XhoI (Thermo Fisher Scientific) restriction enzyme recognition sites at 3′ and 5′ termini, the primer pairs utilized to amplify 3′-UTR segments were synthesized. Cloning 3′-UTR segment occurred within the psiCHECK2 vector (Promega). To identify the clones including PTEN 3′-UTR and miR-183, colony PCR was utilized.

### Luciferase activity assays

The impacts of miR-183 on the 3′-UTR activity of PTEN were assessed through a dual luciferase assay in the HEK-293T cell. To seed the HEK-293T cell, 48-well plates were considered in triplicate. 3′UTR sequence of Wnt-7b was used as an off-target in the assay. Wnt-7b 3′-UTR or PTEN 3′-UTR were co-transfected with negative mock control or miR-183, utilizing Turbofect transfection reagent based on the manufacturer’s process. Cells were gathered at 48 h post-transfection. Then, a dual-luciferase reporter assay kit (Promega) was used to measure luciferase activity, which was recorded via a chemiluminescence meter.

### Quantitative real-time polymerase chain reaction

After 48 h of the transfection, using TRIzol reagent (Invitrogen), the total RNA was isolated from cells based on the instructions of the manufacturer. A NanoDrop Spectrophotometer was used to assess the quality of RNA from every group of Cells before synthesizing the complementary DNA (cDNA) via a Reverse Transcription Kit (QIAGEN). Based on the amplification instructions, the synthesized cDNA was diluted for quantitative real-time polymerase chain reaction (qRT-PCR) with SYBR Green Real-time PCR Master Mix (Thermo Fisher Scientific). The 2^-ΔΔCt^ method was used to analyze the expression of miR-183 and its target, PTEN. U48 and GAPDH as housekeeping genes were used. Table 1 presents the used primers.

**Table 1:** Primers used in this study.

Primers	Sequence of primers
miR-183-F	5-GACTATGGCACTGGTAGAATTCA-3
Universal	5-GCGTCGACTAGTACAACTCAAG-3
U48-F	5-TGATGACCCCAGGTAACTCTG-3
Universal	5-GCGTCGACTAGTACAACTCAAG-3
PTEN-F	5-CACACGACGGGAAGACAAGT-3
PTEN-R	5-TCCTCTGGTCCTGGTATGAAG-3
GAPDH-F	5-ACCCACTCCTCCACCTTTGAC-3
GAPDH-R	5-TGTTGCTGTAGCCAAATTCGTT-3
PTEN-3UTR-XhoI-F	5-AATTATCTCGAGAGAATTTGACAAGAATTGCTATGAC-3
PTEN-3UTR-NotI-R	5-AATTAAGCGGCCGCGTCCCACTGTAACATTGTCTACT-3
Pre-miR-183- XhoI-F	5-AATTATCTCGAGCATGTGGATCTTGTGAAGAGGTG-3
Pre-miR-183-HindIII-R	5-AATTAAAAGCTTCTTGCATCCCTGCACCCTTG-3

### In-vitro scratch wound healing assay

Using the wound-healing assay, the transfected BC cells’ horizontal migration capacity was investigated. The cell monolayer was wounded with 200 μL pipette tips in a scratch assay, following transfection. Then, it was photographed under a microscope at 0 and 48 h. To measure the gap size, the ImageJ software was used and the percentage of migration was determined considering the wound size at 0 h.

### Trans-well migration assay

Using the Trans-well assay along with Trans-well inserts (8 μm, 24-well plate, Sigma-Aldrich), the transfected BC cells’ migration capability was analyzed. The cells suspended in DMEM with no serum were seeded in the upper chamber 48 h following transfection. DMEM comprising 10% FBS was inserted into the lower chamber. Cells that had migrated through the pores were fixed and stained with 95% ethanol, and 0.1% crystal violet dye (Sigma-Aldrich) for 15 min respectively. Then, they were counted under a microscope.

### The cell cycle and MTT assay

MCF-7 and MDA-MB-231 cells were transfected with miR-183, and un-transfected cells were used as control. After 48 h, the cells were harvested and stained via propidium iodide (PI, Sigma). After staining with propidium iodide (PI) for 30 min in the dark at 4 °C, a FACScan flow cytometer (Biosciences) was used to analyze the cells. Moreover, an MTT assay was used to determine cell viability. Briefly, seeding the cells in a 96-well plate at a density of 5000 cells/well, they were transfected with miR-183. The culture medium was removed after 48 h of transfection of BC cells with miR-183. Then, 10 µL of sterile MTT (5 mg/mL; Sigma Aldrich) was inserted into each well. After incubation for another 4 h at 37 °C, the MTT solution was eliminated, and 150 μL of dimethyl sulfoxide (DMSO, Sigma-Aldrich) was added. Formazan crystals were dissolved in a shaker for 10 min. The absorbance was determined at 570 nm on a microplate reader (Bio-Rad Laboratories). The blanks were the wells without cells.

### Western blots

At 48 h following transfection, the total protein was extracted from the cells using RIPA buffer (Roche, Nutley, NJ, USA). BCA Protein Assay Kit (Thermo Fisher Scientific) was used to determine the protein concentrations and SDS-PAGE was used to separate proteins, which were then transferred onto PVDF membranes (Millipore, Billerica, MA, USA). Using 5% non-fat dry milk at room temperature for 1 h, the membranes were blocked and then incubated with primary antibodies against PTEN or β-actin (Cell Signaling Technology) at 4 °C overnight. After that, it interacted with horseradish peroxidase-conjugated secondary antibodies (Cell SignalingTechnology) at room temperature for 2 h. An enhanced chemiluminescence (ECL) system was used to visualize all blots.

### Statistical analysis

To perform statistical analysis, Graph Pad Prism v.8.4.3 was used. The results were expressed as mean ± standard deviation (SD). Using the student's t-test, comparisons between the two experimental groups were performed. One-way ANOVA was used to assess the significance of differences among three groups or more. A p < 0.05 was a significant difference statistically.

## Supplementary Information

Supplementary Information 1.

Supplementary Information 2.

## Data Availability

The datasets analyzed during the current study are available in the (GSE68085)/https://www.ncbi.nlm.nih.gov/Traces/study/?acc=PRJNA281709&o=acc_s%3Aa and (GSE117452)/https://www.ncbi.nlm.nih.gov/Traces/study/?acc=PRJNA482141&o=acc_s%3Aa.
