# Supplementary material for: Mir-183 functions as an oncogene via decreasing PTEN in breast cancer cells
Source: Sci Rep. 2023 May 19;13:8086. doi: 10.1038/s41598-023-35059-x (PMC10199038; doi:10.1038/s41598-023-35059-x)
Supplement: Supplementary file 1 — Supplementary Information 1. [file 41598_2023_35059_MOESM1_ESM.pdf]

1. GSE117452

Tumor	Normal
1. GSM3295068 9648	1. GSM3295097 normal-1
2. GSM3295069 12777	2. GSM3295098 normal-2
3. GSM3295070 13014	3. GSM3295099 normal-5
4. GSM3295071 14503	4. GSM3295121 normal-4-lcc28105
5. GSM3295072 14843	5. GSM3295122 normal-6-lcc18618
6. GSM3295073 14994	6. GSM3295123 normal-7-lcc17644
7. GSM3295074 15362	7. GSM3295132 normal-10-lcc17322
8. GSM3295075 15980	8. GSM3295133 normal-11-lcc21258
9. GSM3295076 16053	9. GSM3295134 normal-12-lcc27086
10. GSM3295077 17469	10. GSM3295135 normal-8-lcc13694
11. GSM3295078 18367	
12. GSM3295079 18664	
13. GSM3295080 8785	
14. GSM3295081 10820	
15. GSM3295082 14045	
16. GSM3295083 14128	
17. GSM3295084 14535	
18. GSM3295085 14738	
19. GSM3295086 16172	
20. GSM3295087 16296	
21. GSM3295088 16722	
22. GSM3295089 17506	
23. GSM3295090 18033	
24. GSM3295091 18612	
25. GSM3295092 12151	
26. GSM3295093 16823	
27. GSM3295094 17453	
28. GSM3295095 17982	
29. GSM3295096 19282	
30. GSM3295100 9038	
31. GSM3295101 11119	
32. GSM3295102 13240	
33. GSM3295103 13558	
34. GSM3295104 14302	
35. GSM3295105 17074	
36. GSM3295106 17098	
37. GSM3295107 17614	
38. GSM3295108 17812	
39. GSM3295109 18475	
40. GSM3295110 18800	

41. GSM3295111 18986<br>42. GSM3295112 11326<br>43. GSM3295113 12694<br>44. GSM3295114 14496<br>45. GSM3295115 14677<br>46. GSM3295116 17271<br>47. GSM3295117 18313<br>48. GSM3295118 18534<br>49. GSM3295119 19011<br>50. GSM3295120 20376<br>51. GSM3295124 9048<br>52. GSM3295125 13913<br>53. GSM3295126 14051<br>54. GSM3295127 14308<br>55. GSM3295128 14944<br>56. GSM3295129 17411<br>57. GSM3295130 18268<br>58. GSM3295131 19268	

2. GSE68085

Tumor	Normal
1. GSM1662431 BM01-13: Tumor tissue<br>2. GSM1662432 BM01-15: Tumor tissue<br>3. GSM1662433 BM01-27: Tumor tissue<br>4. GSM1662434 BM01-29: Tumor tissue<br>5. GSM1662435 BM01-34: Tumor tissue<br>6. GSM1662436 BM01-5: Tumor tissue<br>7. GSM1662437 C1010: Tumor tissue<br>8. GSM1662438 C108: Tumor tissue<br>9. GSM1662439 C131: Tumor tissue<br>10. GSM1662440 C135: Tumor tissue<br>11. GSM1662441 C139: Tumor tissue<br>12. GSM1662442 C140: Tumor tissue<br>13. GSM1662443 C152: Tumor tissue<br>14. GSM1662444 C165: Tumor tissue<br>15. GSM1662445 C166: Tumor tissue<br>16. GSM1662446 C18: Tumor tissue	1. GSM1662420 MT1253: Normal tissue<br>2. GSM1662421 MT1261: Normal tissue<br>3. GSM1662422 MT1273: Normal tissue<br>4. GSM1662423 MT1299: Normal tissue<br>5. GSM1662424 MT1345: Normal tissue<br>6. GSM1662425 MT1451: Normal tissue<br>7. GSM1662426 MT1491: Normal tissue<br>8. GSM1662427 MT1537: Normal tissue<br>9. GSM1662428 MT4974_V04: Normal tissue<br>10. GSM1662429 MT5048_V02: Normal tissue

17. GSM1662447 C197: Tumor tissue<br>18. GSM1662448 C201: Tumor tissue<br>19. GSM1662449 C203: Tumor tissue<br>20. GSM1662450 C204: Tumor tissue<br>21. GSM1662451 C22: Tumor tissue<br>22. GSM1662452 C225: Tumor tissue<br>23. GSM1662453 C229: Tumor tissue<br>24. GSM1662454 C23: Tumor tissue<br>25. GSM1662455 C234: Tumor tissue<br>26. GSM1662456 C249: Tumor tissue<br>27. GSM1662457 C250: Tumor tissue<br>28. GSM1662458 C257: Tumor tissue<br>29. GSM1662459 C258: Tumor tissue<br>30. GSM1662460 C268: Tumor tissue<br>31. GSM1662461 C280: Tumor tissue<br>32. GSM1662462 C287: Tumor tissue<br>33. GSM1662463 C288: Tumor tissue<br>34. GSM1662464 C304: Tumor tissue<br>35. GSM1662465 C306: Tumor tissue<br>36. GSM1662466 C336: Tumor tissue<br>37. GSM1662467 C340: Tumor tissue<br>38. GSM1662468 C342: Tumor tissue<br>39. GSM1662469 C363: Tumor tissue<br>40. GSM1662470 C389: Tumor tissue<br>41. GSM1662471 C440: Tumor tissue<br>42. GSM1662472 C451: Tumor tissue<br>43. GSM1662473 C49: Tumor tissue<br>44. GSM1662474 C494: Tumor tissue<br>45. GSM1662475 C521: Tumor tissue<br>46. GSM1662476 C533: Tumor tissue<br>47. GSM1662477 C537: Tumor tissue<br>48. GSM1662478 C56: Tumor tissue<br>49. GSM1662479 C586: Tumor tissue<br>50. GSM1662480 C60: Tumor tissue<br>51. GSM1662481 C702: Tumor tissue<br>52. GSM1662482 C704: Tumor tissue<br>53. GSM1662483 C75: Tumor tissue<br>54. GSM1662484 C81: Tumor tissue<br>55. GSM1662485 CT74: Tumor tissue<br>56. GSM1662486 GT07: Tumor tissue<br>57. GSM1662487 GT141: Tumor tissue<br>58. GSM1662488 GT226: Tumor tissue<br>59. GSM1662489 GT74: Tumor tissue<br>60. GSM1662490 GT91: Tumor tissue<br>61. GSM1662491 GT93: Tumor tissue<br>62. GSM1662492 MT1023: Tumor tissue	11. GSM1662430 MT5049_V02: Normal tissue

63. GSM1662493 MT1029: Tumor tissue<br>64. GSM1662494 MT111: Tumor tissue<br>65. GSM1662495 MT1118: Tumor tissue<br>66. GSM1662496 MT1213: Tumor tissue<br>67. GSM1662497 MT1230: Tumor tissue<br>68. GSM1662498 MT1266: Tumor tissue<br>69. GSM1662499 MT1275: Tumor tissue<br>70. GSM1662500 MT1310: Tumor tissue<br>71. GSM1662501 MT1315: Tumor tissue<br>72. GSM1662502 MT1337: Tumor tissue<br>73. GSM1662503 MT1386: Tumor tissue<br>74. GSM1662504 MT1525: Tumor tissue<br>75. GSM1662505 MT1584: Tumor tissue<br>76. GSM1662506 MT1643: Tumor tissue<br>77. GSM1662507 MT174: Tumor tissue<br>78. GSM1662508 MT1794: Tumor tissue<br>79. GSM1662509 MT1847: Tumor tissue<br>80. GSM1662510 MT196: Tumor tissue<br>81. GSM1662511 MT219: Tumor tissue<br>82. GSM1662512 MT237: Tumor tissue<br>83. GSM1662513 MT2385: Tumor tissue<br>84. GSM1662514 MT265: Tumor tissue<br>85. GSM1662515 MT269: Tumor tissue<br>86. GSM1662516 MT290: Tumor tissue<br>87. GSM1662517 MT350: Tumor tissue<br>88. GSM1662518 MT359: Tumor tissue<br>89. GSM1662519 MT450: Tumor tissue<br>90. GSM1662520 MT469: Tumor tissue<br>91. GSM1662521 MT518: Tumor tissue<br>92. GSM1662522 MT53: Tumor tissue<br>93. GSM1662523 MT578: Tumor tissue<br>94. GSM1662524 MT658: Tumor tissue<br>95. GSM1662525 MT689: Tumor tissue<br>96. GSM1662526 MT75: Tumor tissue<br>97. GSM1662527 MT750: Tumor tissue<br>98. GSM1662528 MT77: Tumor tissue<br>99. GSM1662529 MT79: Tumor tissue<br>100. GSM1662530 MT813: Tumor<br>101. GSM1662531 MT895: Tumor<br>102. GSM1662532 MT979: Tumor<br>103. GSM1662533 MT990: Tumor	
