# Supplementary material for: Mir-183 functions as an oncogene via decreasing PTEN in breast cancer cells
Source: Sci Rep. 2023 May 19;13:8086. doi: 10.1038/s41598-023-35059-x (PMC10199038; doi:10.1038/s41598-023-35059-x)
Supplement: Supplementary file 2 — Supplementary Information 2. [file 41598_2023_35059_MOESM2_ESM.pdf]

Mir-183 functions as an oncogene via decreasing PTEN in breast cancer cells

Samaneh Mohammaddoust[1], Majid Sadeghizadeh[1*]

*Corresponding author: Majid Sadeghizadeh; Email: Sadeghma@modares.ac.ir

1. Genetics Department, Faculty of Biological Sciences, Tarbiat Modares University, Tehran, Iran

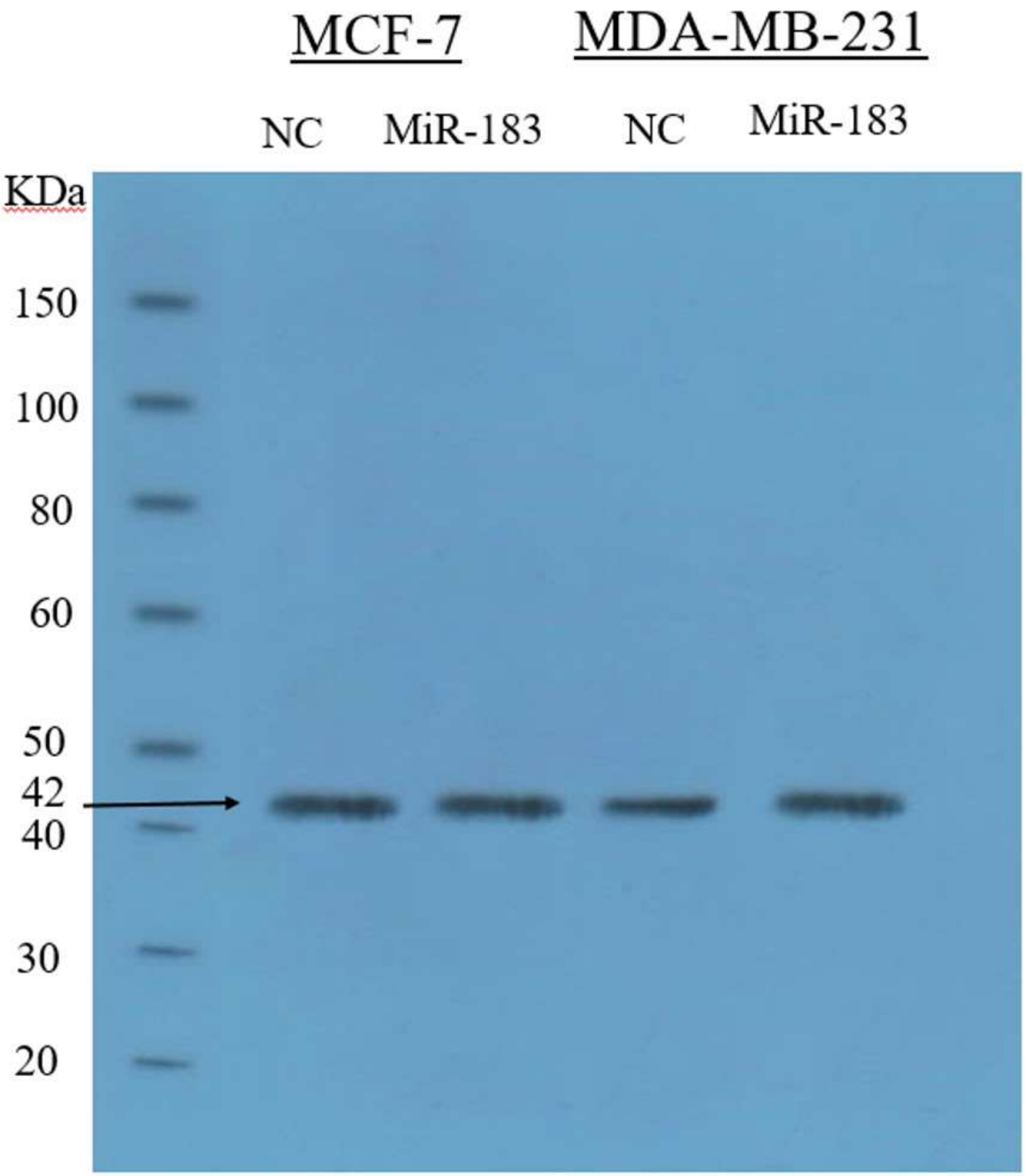

Fig1. Western blotting of the **Actin.** Lane1: protein molecular weight marker, Lane2: NC in MCF-7, Lane3: MiR-183 overexpression in MCF-7, Lane4: NC in MDA-MB-231, Lane5: MiR-183 overexpression in MDA-MB-231.

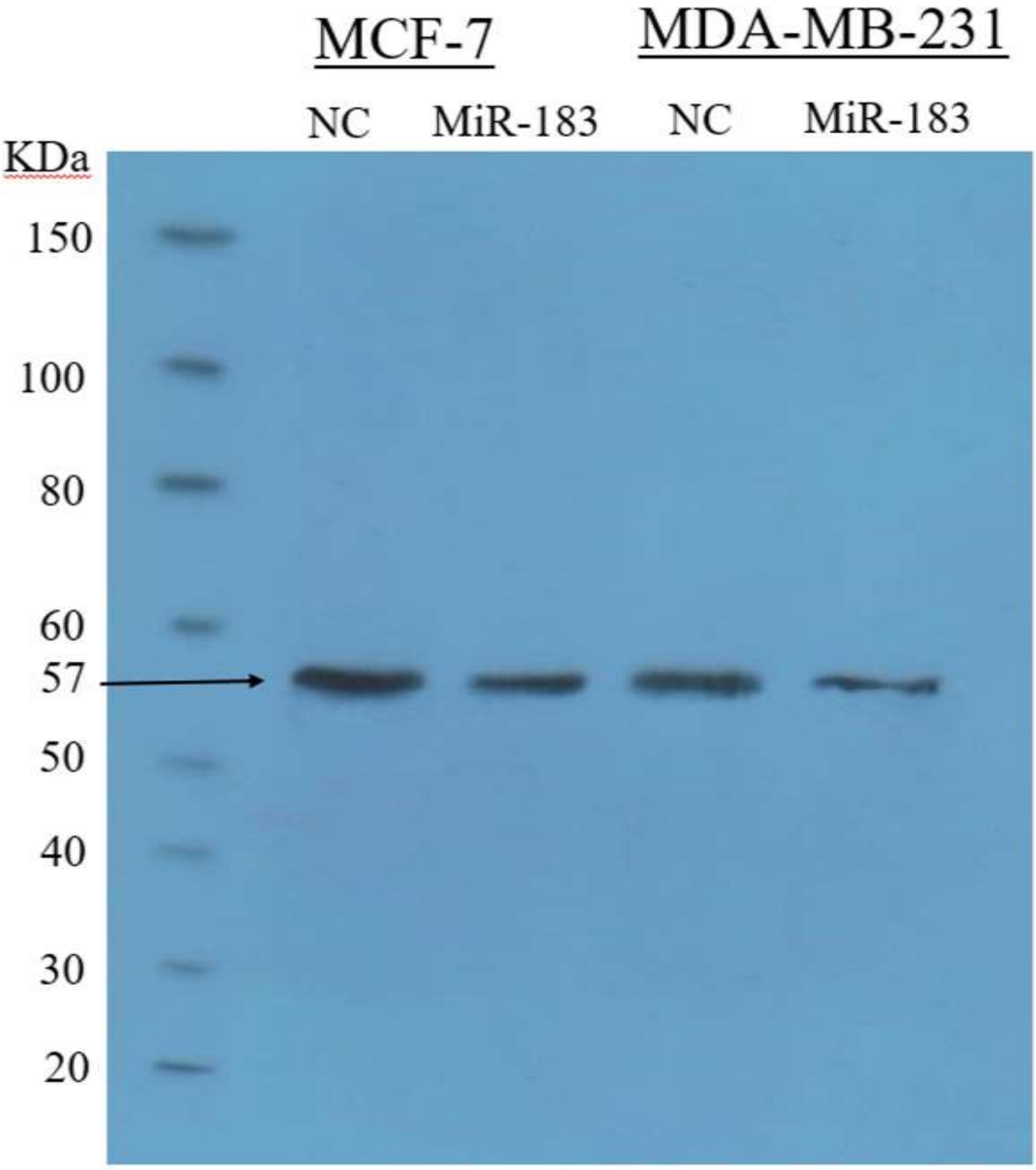

Fig2. Western blotting of the **PTEN**. Lane1: protein molecular weight marker, Lane2: NC in MCF-7, Lane3: MiR-183 overexpression in MCF-7, Lane4: NC in MDA-MB-231, Lane5: MiR-183 overexpression in MDA-MB-231.
